# Secondary Metabolites from Marine-Derived Bacteria with Antibiotic and Antibiofilm Activities against Drug-Resistant Pathogens †

**DOI:** 10.3390/md21010050

**Published:** 2023-01-12

**Authors:** Joko Tri Wibowo, Asep Bayu, Widya Dwi Aryati, Carla Fernandes, Arry Yanuar, Anake Kijjoa, Masteria Yunovilsa Putra

**Affiliations:** 1 Research Center for Vaccine and Drug, Research Organization for Health, National Research and Innovation Agency (BRIN), KST Soekarno Jl. Raya Bogor Km. 46, Cibinong 16911, Indonesia; 2 Faculty of Pharmacy, Universitas Indonesia, Depok 16424, Indonesia; 3 Laboratório de Química Orgânica e Farmacêutica, Departamento de Ciências Químicas, Faculdade de Farmácia, Universidade do Porto and CIIMAR, Rua de Jorge Viterbo Ferreira 228, 4050-313 Porto, Portugal; 4 National Metabolomics Collaborative Research Center, Faculty of Pharmacy, Universitas Indonesia, Kampus UI Depok, Depok 16424, Indonesia; 5 ICBAS-Instituto de Ciências Biomédicas Abel Salazar and CIIMAR, Universidade do Porto, Rua de Jorge Viterbo Ferreira 228, 4050-313 Porto, Portugal

**Keywords:** marine-derived bacteria, drug-resistant pathogens, novel antibiotics, secondary metabolites

## Abstract

The search for new antibiotics against drug-resistant microbes has been expanded to marine bacteria. Marine bacteria have been proven to be a prolific source of a myriad of novel compounds with potential biological activities. Therefore, this review highlights novel and bioactive compounds from marine bacteria reported during the period of January 2016 to December 2021. Published articles containing novel marine bacterial secondary metabolites that are active against drug-resistant pathogens were collected. Previously described compounds (prior to January 2016) are not included in this review. Unreported compounds during this period that exhibited activity against pathogenic microbes were discussed and compared in order to find the cue of the structure–bioactivity relationship. The results showed that *Streptomyces* are the most studied bacteria with undescribed bioactive compounds, followed by other genera in the Actinobacteria. We have categorized the structures of the compounds in the present review into four groups, based on their biosynthetic origins, as polyketide derivatives, amino acid derivatives, terpenoids, as well as compounds with mixed origin. These compounds were active against one or more drug-resistant pathogens, such as methicillin-resistant *Staphylococcus aureus* (MRSA), methicillin-resistant *Staphylococcus epidermidis* (MRSE), vancomycin-resistant *Enterococci* (VRE), multidrug-resistant *Mycobacterium tuberculosis* (MDR-TB), and amphotericin B-resistant *Candida albicans*. In addition, some of the compounds also showed activity against biofilm formation of the test bacteria. Some previously undescribed compounds, isolated from marine-derived bacteria during this period, could have a good potential as lead compounds for the development of drug candidates to overcome multidrug-resistant pathogens.

## 1. Introduction

One of the most significant global concerns regarding health issues is the emergence and rapid spread of drug-resistant pathogens that have acquired new resistance mechanisms, leading to antimicrobial resistance [1]. Currently, the pan- or multidrug-resistant (MDR) microbes are responsible for about 40–60% of the infection cases in many low- to middle-income countries, such as Indonesia, Brazil, and Russia [2]. It is predicted that antibiotic resistance in these countries will rise four to seven times faster than in other 37 member countries of the Organization for Economic Co-operation and Development (OECD) [2]. The immense burden caused by the drug-resistant microorganisms affects not only human health but also the economy. The report by OECD in 2018 showed that around 2.4 million individuals could die in Europe, North America, and Australia from 2015 to 2050 due to antimicrobial resistance (AMR). AMR would cost about 3.5 billion USD purchasing power parity (PPP) per year to the healthcare services of this group of countries [2].

The mechanisms through which bacteria develop resistance to antibiotics can be due to either their intrinsic resistance to antibiotics or their ability to develop/acquire antibiotic resistance genes. Intrinsic ability of bacteria to resist a particular antibiotic is a result of inherent structural or functional characteristics [3]. Acquired resistance can occur through vertical evolution (including mutation and selection) or horizontal evolution from genetic exchange with other bacteria (including transformation, conjugation, or transduction) [4]. Bacteria may also develop adaptive resistance to one or more antibiotics through induction by a specific signal or environmental cues. The resulting transient adaptive resistance is not vertically inherited but will be back to the original state upon removal of the signal or environmental cues [5]. 

There are quite a few antibiotics that are produced by bacteria, especially from actinomycetes (*Streptomyces*), such as streptomycin, tetracycline, chloramphenicol, erythromycin, viomycin, lincomycin, meropenem, and daptomycin. Nonetheless, antibiotics are also produced from other phyla of bacteria, such as gramicidin A from *Bacillus*, colistin from *Paenibacillus*, mupirocin from *Pseudomonas*, and aztreonam, a monobactam antibiotic, which was obtained by synthesis based on the structure of the compound produced by *Chromobacterium violaceum* [6]. Intriguingly, although the emergence of antibiotic-resistant pathogens has stimulated a renewed effort to search for new antibiotics with unique structures and different mechanisms of action from actinomycetes or related taxa, this endeavor often leads to the rediscovery of the already known antibiotics [7].

In recent years, the interest in finding novel antibiotic-producing bacteria has shifted from terrestrial strains to their marine counterparts due to the following reasons: (i) The marine environment is considered an underexplored source, (ii) high-stress environment makes the marine-derived bacteria produce metabolites different from their terrestrial counterparts, and (iii) the advancement of technologies for bioprospection that can allow the collection of samples from deep sea, thermal vent, or polar region [8,9,10]. Potential secondary metabolites from marine bacteria with strong antibiotic activity against drug-resistant pathogens have been reviewed by Schinke et al. [11], covering the compounds obtained from marine-derived bacteria during the period of 2010–2015.

The present review discusses the structures of undescribed secondary metabolites with antibiotic/antibiofilm activities as well as the producing organisms, reported from January 2016 to December 2021, highlighting the metabolites that are active against drug-resistant pathogenic bacteria, such as *Enterococcus faecium*, *Staphylococcus aureus*, *Klebsiella pneumoniae*, *Acinetobacter baumannii*, *Pseudomonas aeruginosa*, and *Enterobacter* species (ESAKPE).

## 2. Secondary Metabolites from Marine-Derived Bacteria Which Were Tested against Drug-Resistant Bacteria

In order to facilitate readers’ comprehension, secondary metabolites isolated from marine-derived bacteria, which were tested against drug-resistant pathogens during the period of January 2016 to December 2021, were classified according to their biosynthetic origins in the first level. Then, each group was divided into subgroups according to their chemical scaffolds.

### 2.1. Polyketides 

#### 2.1.1. Anthraquinones and Their Analogs

**Figure 1:**
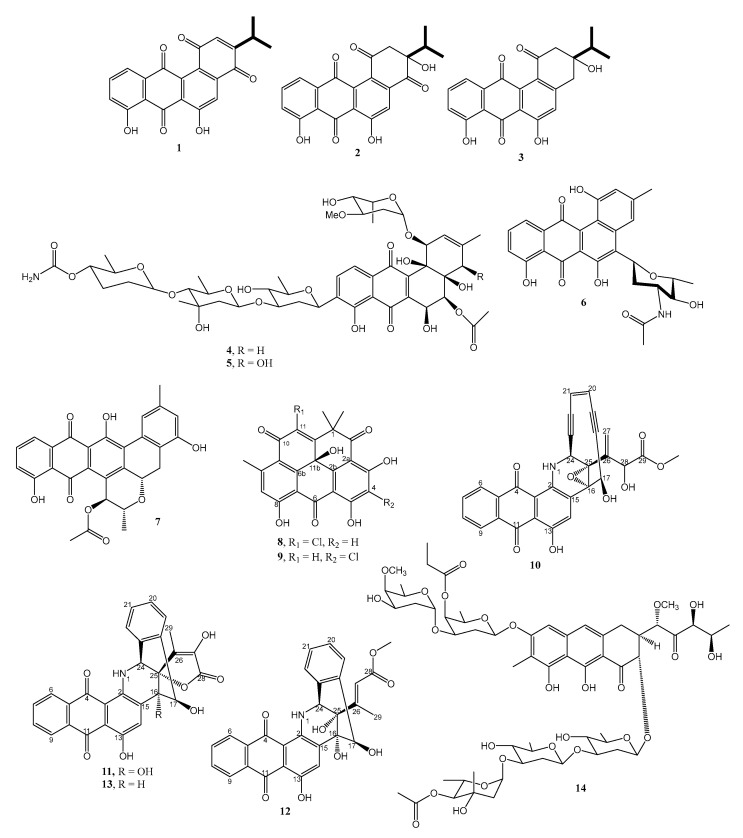
Structure of **1**–**14**.

Three unreported angucycline-type aromatic polyketides, nocardiopsistins A-C (**1**–**3**) (Figure 1), were isolated from a deep-sea actinobacterium, *Nocardiopsis* sp. strain HB-J378. The production of these compounds was significantly increased with the addition of lanthanum chloride (LaCl_3_) to the SPY medium (39.5 g of sea salt, 20 g of soluble starch, 10 g of glucose, 5 g of peptone, 5 g of yeast extract, 2 g of CaCO_3_, 0.5 g of K_2_HPO_4_, and 0.5 g of MgSO_4_·7H_2_O per liter). Incubation of the bacterium in the SPY medium was done at 28 °C for 7 days. The chemical structures of **1**–**3** were established using interpretation of 1D and 2D NMR as well as high-resolution electrospray ionization mass spectrometry (HRESIMS) data. Compounds **1**–**3** were active against methicillin-resistant *Staphylococcus aureus* (MRSA), with minimum inhibitory concentration (MIC) values of 12.5, 3.12, and 12.5 μg/mL, respectively. Interestingly, **2** exhibited the same MIC value as chloramphenicol, a positive control used in this study [12].

Two undescribed angucycline-type glycosides, namely stremycins A (**4**) and B (**5**) (Figure 1), were isolated from the culture extract of *Streptomyces pratensis* strain NA-ZhouS1, which was obtained from a marine sediment in Zhoushan, East China Sea [13]. The structures of both compounds were determined using high-resolution time-of-flight mass spectrometry (HR-TOF-MS), 1D, and 2D NMR techniques. Compounds **4** and **5** have the same glycone moiety, comprising β-olivose-(4→1)-β-olivomycose-(3→1)-4-*O*-carbamoyl-β-amicetose, but slightly differ only in the tetracyclic benz[α]anthracene skeleton with the hydroxyl group on C-4 in **5**. Both compounds were isolated from 25 L of a metal-stress medium culture (liquid gauze’s medium containing 20 g of soluble starch, 1 g of KNO_3_, 0.5 g of K_2_HPO_4_, 0.5 g of MgSO_4_·7H_2_O, 0.01 g of FeSO_4_·7H_2_O, 35 g of sea salt per liter at pH 7.4 and supplemented with 100 µM of NiCl_2_·6H_2_O) and incubated at 28 °C for 10 days. Nickel was selected due to its capacity to activate cryptic gene clusters, which induce the production of bioactive metabolites that were not detected in the medium without metal. Both compounds showed antibacterial activity with a MIC value of 16 µg/mL against *P. aeruginosa*, methicillin-resistant *S. aureus* (MRSA), *K. pneumoniae*, and *E. coli*. When tested against *B. subtilis*, both compounds showed MIC values of 8–16 µg/mL. Therefore, the variation in the aglycone part does not significantly alter antibacterial bioactivities.

The marine sediment-derived actinomycete, *Streptomyces* sp. 182SMLY, yielded polycyclic anthraquinone compounds, namely *N*-acetyl-*N*-demethylmayamycin (**6**) and streptoanthraquinone A (**7**) (Figure 1). Compounds **6** and **7** were isolated from the fermentation of the bacterium at 28 °C for 7 days in the Gause’s liquid medium (20 g/L starch, 1.0 g/L KNO_3_, 0.5 g/L K_2_HPO_4_, 0.5 g/L MgSO_4_·7H_2_O, 0.5 g/L NaCl, and 0.01 g/L FeSO_4_·7H_2_O). The structure of **6** was established using 1D and 2D NMR, HRESIMS techniques. The relative configurations of the stereogenic carbons of the amino sugar moiety were determined by NOESY correlations, while their absolute configurations were established by comparison of the experimental and calculated electronic circular dichroism (ECD) spectra. The antibiotic activities of **6** and **7** were tested against methicillin-resistant *S. aureus* ATCC 43300 and *E. coli* ATCC 25922, however, **7** was not active. Compound **6** showed inhibition activity only against MRSA with a MIC value of 20.0 µM. The positive control, norfloxacin, exhibited MIC values of 62.6 µM against both *E. coli* and MRSA [14].

Two unreported resistoflavin derivatives, chlororesistoflavins A (**8**) and B (**9**) (Figure 1), were obtained from the culture extract of *Streptomyces* sp. strain EG32, isolated from a sediment collected from the northern coast of Egypt (Mediterranean Sea) and cultured in the Waksman liquid medium (20.0 g glucose, 5.0 g peptone, 5.0 g beef extract, 3.0 g yeast extract, 3.0 g CaCO_3_, 5.0 g NaCl, 50% seawater, 50% DI H_2_O, 10 L in total) at 27 °C for 7 days. The structures of both compounds were established by extensive 1D and 2D NMR and HR-ESI-TOF-MS spectral analysis. Intriguingly, even though **8**, **9,** and resistoflavin, all possessing only one stereogenic carbon (C-11b), displayed the same sign of optical rotation (levorotatory) and shared the same biosynthetic origin, the ECD spectrum of **8** exhibited a strong Cotton effect near the n-π* transition of the carbonyl at its C-10, which is antipodal to the ECD spectra of resistoflavin and **9**. The authors hypothesized that a chlorine atom imposes severe allylic-1,3 strain in **8**, distorting the ring from the ideal conformation found in **9** and resistoflavin, thus altering the first-sphere contributions to the Cotton effect. By using molecular mechanics (MM) and density functional theory (DFT) calculations of both **8** and **9**, they have found that **8** contained a cyclohexenone ring that has a severely distorted conformation from that of **9** and resistoflavin by steric influence of the bulky, electron-rich chlorine atom on C-11 and the carbonyl group at C-10. The conformational ring bending of the cyclohexenone in **8**, when compared to **9**, is interpreted as forcing the disposition of the heavy chlorine substituent from a negative-contributing quadrant to a positive-contributing quadrant. Therefore, the authors concluded that the absolute configuration of C-11b in **8** is the same as that of C-11b in **9** and resistoflavin, i.e., *R* configuration.

Compound **8** showed comparable inhibitory activity to resistoflavin (MIC = 0.25 µg/mL) but eight times stronger than **9** (MIC = 2.0 µg/mL) against MRSA. The positive control, ciprofloxacin, showed a MIC value of 0.2 µg/mL against MRSA. Interestingly, the position of the chlorine substituent on the resistoflavin scaffold was found to affect the activity of its analogs. For instance, when the chlorine atom is on C-11, as in **8**, its activity is comparable to that of the parent compound, i.e., resistoflavin. However, when the chlorine atom is on C-4, as in **9**, its activity is reduced when compared to resistoflavin [15]. 

The liquid culture of *Nonomuraea* sp. strain MM565M-173N2, isolated from a deep-sea sediment collected in Japan trench at a depth of 329 m off the Sanriku coast, furnished sealutomicins A-D (**10**–**13**) (Figure 1). Compounds **10**–**13** were obtained from 220 L of the fermented production medium containing 0.75% glycerin, 0.75% cotton seed meal, 0.25% L-glutamate, 0.15% NaCl, and 1.8% Daigo’s Artificial Seawater SP (pH value 7.4 before sterilization). The fermentation was performed at 27 °C for 7 days on a rotary shaker (180 rpm). The structures of **10**–**13** were established by extensive analysis of their 1D and 2D NMR and HRESIMS spectra. In the case of **10**, the nuclear Overhauser effect (NOE) data revealed the relative configurations as 16S*, 17R*, 24S*, 25S*. Since its circular dichroism (CD) spectrum is nearly identical to that of a related compound, dynemicin A, its absolute stereochemistry was established as 16*S*, 17*R*, 24*S*, 25*S*. However, the absolute configuration of C-28 of the side chain could not be determined. The relative configurations of the stereogenic carbons of **11**–**13** were established by NOE data, whereas their absolute configurations were determined by comparison of their CD spectra with that of **10**. Since **11**–**13** were products from the Bergman cyclization of **10**, which retain the stereochemistry at C-16, C-24, and C-25, the absolute structures of **11**–**13** were determined. 

Compound **10** displayed strong antimicrobial activities against susceptible and MDR Gram-negative bacteria, including New Delhi metallo-beta-lactamase (NDM) and *K. pneumoniae* carbapenemase (KPC)-producing strains, with MIC values of 0.05–0.1 μg/mL for *E. coli*, and 0.1–0.4 μg/mL for *K. pneumoniae*. Moreover, **10** showed potent antimicrobial activities against susceptible and MDR Gram-positive bacteria, including methicillin-resistant *S. aureus* (MRSA) and vancomycin-resistant Enterococci (VRE) strains, with MIC values of 0.00625–0.0125 μg/mL for *S. aureus*, and 0.025–0.1 μg/mL for *Enterococcus faecalis*/*faecium*. Compounds **11**–**13** exhibited moderate to strong antibacterial activity against Gram-positive bacteria, with MIC values of 0.2–1.6 μg/mL against susceptible and MDR *S. aureus*, and 0.8 to more than 6.4 μg/mL for susceptible and *E. faecalis/faecium* VRE. However, they did not show activity against Gram-negative bacteria. Meropenem, a positive control, showed MIC values ranging from 0.1–6.4 µg/mL. The positive controls, meropenem, showed MIC values ranging from 0.0125 μg/mL (toward *E. coli* K-12) to 6.4 μg/mL (toward *S. aureus* MRSA.), while colistin displayed MIC values ranging from 0.4 μg/mL (toward *E. coli* K-12) to 6.4 μg/mL (toward *E. coli* MCR) [16].

Antibacterial activity-guided isolation of a marine sediment-derived bacterium, *Streptomyces* sp. MBTI36, yielded an undescribed glycosylated 6,8,9-trihydroxy-3,4-dihydroanthracen-1 (2*H*)-one derivative, chromomycin A_9_ (**14**) (Figure 1). Analysis of the 16S rRNA of the bacterium showed 99.9% sequence similarity to *Streptomyces microflavus* NBRC13062. Compound **14** was isolated from the fermentation of the bacterium at 28 °C for 14 days without shaking in a total of 20 L of the liquid GTYB medium (each liter of the medium contains 10 g of glucose, 2 g of tryptone, 1 g of yeast extract, and 1 g of beef extract in 1 L of artificial seawater). The structure of **14** was established based on 1D and 2D NMR and HRESIMS spectral analysis as well as a comparison of its ^1^H and ^13^C NMR data with the previously reported chromomycins Ap, A_2_, and A_3_. 

Compound **14** displayed potent antibacterial activities against all methicillin-sensitive *S. aureus* (MSSA) and MRSA strains, showing significant broad-spectrum antibiotic effects on MRSA strains with MIC values of 0.06–0.25 µg/mL, which are more potent than some of the major classes of antibiotics, such as daptomycin (MIC > 32 µg/mL), vancomycin (MIC = 0.5–2 µg/mL), platensimycin (MIC = 4–8 µg/mL), linezolid (MIC = 1–2 µg/mL), and ciprofloxacin (MIC = 0.13 > 32 µg/mL) [17].

*S. aureus* ATCC43300 was selected to evaluate its resistance development against ciprofloxacin and **14**. A steady increase in MIC was observed for ciprofloxacin during the passage experiment in *S. aureus* ATCC43300, with a 128-fold change (MIC = 32 µg/mL) when compared with the initial MIC value (MIC = 0.25 µg/mL). On the contrary, a 2-fold increase in the MIC (from 0.13 to 0.25 µg/mL) was observed for *S. aureus* ATCC43300 after 21 passages. Therefore, during the passage experiment, MIC values for **14** did not increase more than 4-fold from its initial MIC value, confirming that there was no resistance development during the 21 passages [17].

#### 2.1.2. Naphthoquinones

**Figure 2:**
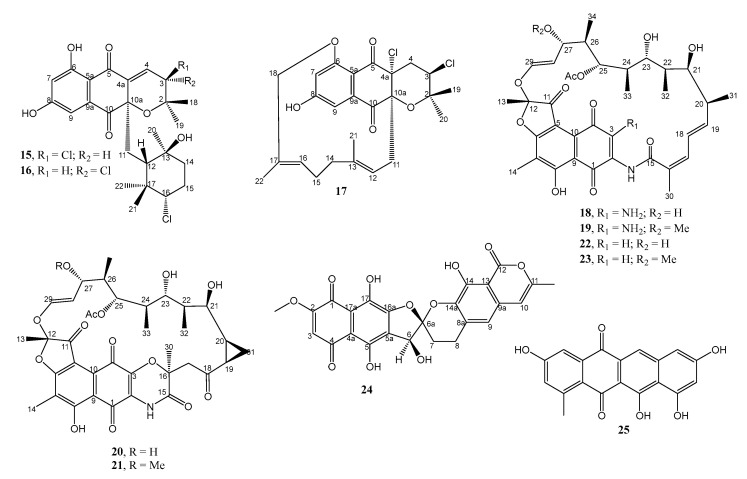
Structures of **15**–**25**.

The undescribed napyradiomycins, i.e., napyradiomycin B7a (**15**), napyradiomycin B7b (**16**), and napyradiomycin D1 (**17**) (Figure 2), were isolated from the EtOAc extract of the culture of *Streptomyces* sp. strain CA-271078 (which showed 99.34% similarity with *Streptomyces aculeolatus* NBRC 14824(T)), fermented at 28 °C in 3 L of the R358 medium for 6 days.

The structures of **15**, **16**, and **17** were established by the interpretation of HR-ESI-TOF-MS, 1D, and 2D NMR spectra. The relative stereochemistry of both compounds was determined based on NOESY and ROESY correlations of the key protons as well as the values of coupling constants of some protons. The absolute configurations of the stereogenic carbons in **15** and **16** were assumed to be the same as those of all the previously reported napyradiomycins in the B series on the basis of their common biosynthetic origin as well as the same signs of their specific rotations. The structure of napyradiomycins can be recognized by the semi-naphthoquinone chromophore, a prenyl unit attached to C-4a, which is cyclized to form a tetrahydropyran ring in most cases, and a monoterpenoid subunit attached to C-10a. Compounds **15** and **17** displayed moderate inhibitory activity against MRSA, with MIC values of 48 and 12–24 µg/mL, respectively. In addition, both compounds also inhibited the growth of *Mycobacterium tuberculosis* H37Ra, with MIC values of 12–24 and 24–48 µg/mL, respectively, while **16** exhibited a MIC value > 64 µg/mL against both MRSA and *M. tuberculosis*. However, the assay did not include any positive control in the experimental design. The results suggested that the absolute configurations at C-3 of **15** and **16** have a significant influence on the antibacterial activity [18].

Bioassay-guided fractionation of the EtOAc extracts of solid agar cultures of *Micromonospora* sp. RJA4480, isolated from a marine sediment obtained at −85 m in Barkley Sound, British Columbia, that exhibited potent in vitro inhibition of MRSA and *E. coli*, led to the identification of four undescribed macrolides containing a naphthoquinone core, 3-amino-27-demethoxy-27-hydroxyrifamycin S (**18**), 3-aminorifamycin S (**19**), sporalactam A (**20**), and sporalactam B (**21**) (Figure 2) as the antibacterial componentes. Although no 3-amino ansa macrolide has been reported as a natural product, **19** has been previously obtained by semisynthesis [19].

The structure of **18** was established by interpretation of HR-ESI-TOF-MS, 1D, and 2D NMR spectral data. Curiously, the proton and carbon signals in the ^1^H and ^13^C NMR spectra of **18**, recorded in deuterated dimethyl sulfoxide (DMSO-*d6*), were doubled. The single-crystal X-ray diffraction analysis of **18** did not only determine its absolute structure as 12*S*, 20*S*, 21*S*, 22*R*, *23R*, 24*R*, 25*S*, 26*S*, 27*S* but also revealed that **18** existed in two conformations, thus causing doubled signals in the ^1^H and ^13^C NMR spectra. The structures of **20** and **21** were established by HRESIMS data and extensive analysis of their 1D and 2D NMR spectra. Interestingly, unlike the ^1^H and ^13^C NMR spectra of **18**, the signals in the ^1^H and ^13^C NMR spectra of **20** and **21** are not doubled. The absolute configurations of the stereogenic carbons in **20** and **21** were assigned as 12*S*, 16*S*, 19*R*, 20*S*, 21*S*, 22*R*, 23*R*, 24*R*, 25*S*, *26S*, *27S*, based on the absolute stereochemistry of **18** and of tolypomycinone, a degradation product of tolypomycin Y which is biogenetically related to **20** and **21**. In vitro antimicrobial assay showed that **18**–**21** inhibited the growth of MRSA with MIC_90_ values of 0.0009, 0.0008, 7.0, and 1.8 µM, respectively. The reference compounds, 27-demethoxy-27-hydroxyrifamycin S (**22**) and rifamycin S (**23**) displayed MIC_90_ values of 0.03 and 0.07 µM, respectively. Compounds **18**–**21** also inhibited the growth of *M. tuberculosis* in vitro, with MIC_90_ values of 0.0009, 0.0008, 0.8, and 0.06 µM, respectively (whereas **22** and **23,** displayed MIC_90_ values of 0.04 and 0.006 µM, respectively), and of *M. tuberculosis* growing intracellularly in macrophage cells, with MIC_90_ value ranging from 0.4 to 30 µM (whereas **22** and **23** displayed MIC_90_ values of 3–10 and 0.07 µM, respectively). When comparing MIC_90_ values of **18** and **19** with those of the reference compounds, **22** and **23,** that lack only the amino substituent on C-3, it was found that the 3-amino substituent significantly enhances the potency of these antibiotics against MRSA, *E. coli*, and *M. tuberculosis* [20].

The previously reported 7,8-dideoxygriseorhodin C (**24**) (Figure 2) was obtained from the EtOAc extract of *Streptomyces* sp. strain 1425S.R.1a.1, isolated from a body tissue homogenate of a gastropod mollusk, *Truncatella guerinii*, collected in Cebu, Philippines, and was cultured in R2A broth (0.2% yeast extract, 1% malt extract, 0.2% glucose, and supplemented with 2% NaCl). Although the ^13^C NMR spectrum of **24** was similar to that of 7,8-dideoxygriseorhodin C in the previous report, in this work, the authors have unambiguously assigned all the ^1^H and ^13^C NMR chemical shift values of **24** by 1D and 2D NMR spectral analysis. Since the absolute configuration at C-6 and C-6a of 7,8-dideoxygriseorhodin C had not been established in the previous report, Miller et al. [21] have attempted to determine the absolute configurations of these stereogenic carbons in **24** by comparison of the calculated and experimental ECD spectra. However, this method could only determine the absolute configuration of C-6 as 6*S* with certainty, while the absolute configuration of C-6a was ambiguous. Finally, a combination of the 3D modeling and the correlations observed in the ROESY spectrum have allowed the authors to establish the absolute configuration of C-6a as 6a*S*. Compound **24** inhibited the growth of *S. aureus* ATCC^®^ 43300™ MRSA, which is resistant to oxacillin and methicillin, with a MIC value of 0.08–0.12 µg/mL. The positive control, oxacillin, showed MIC values of 1.59–6.24 μg/mL. Treatment of ATCC^®^ 43300™ MRSA strain with a combination of **24** and oxacillin at 1xMIC showed the reduction of the individual MICs (MIC of **24** = 0.01–0.02 µg/mL; MIC of oxacillin = 0.02–0.298 µg/mL). Moreover, the combination index (CI) for the combination of **24** and oxacillin at 1xMIC was 0.12–0.24, indicating a synergistic effect between **24** and oxacillin [21].

Mersaquinone (**25**) (Figure 2), an unreported tetracene derivative, was isolated from the extract of a marine-derived *Streptomyces* sp. EG1, obtained from a sediment sample that was collected from the North Coast of the Mediterranean Sea, Egypt, and cultured in the Waksman medium at 28 °C for 7 days. The structure of **25** was established based on an extensive analysis of HRESIMS, IR, 1D, and 2D NMR spectra. Compound **25** inhibited the growth of the methicillin-resistant *S. aureus* (MRSA) strain TCH1516, with a MIC value of 3.36 µg/mL. The positive control, ciprofloxacin, showed a MIC value of 0.93 µM [22].

#### 2.1.3. Macrolides

**Figure 3:**
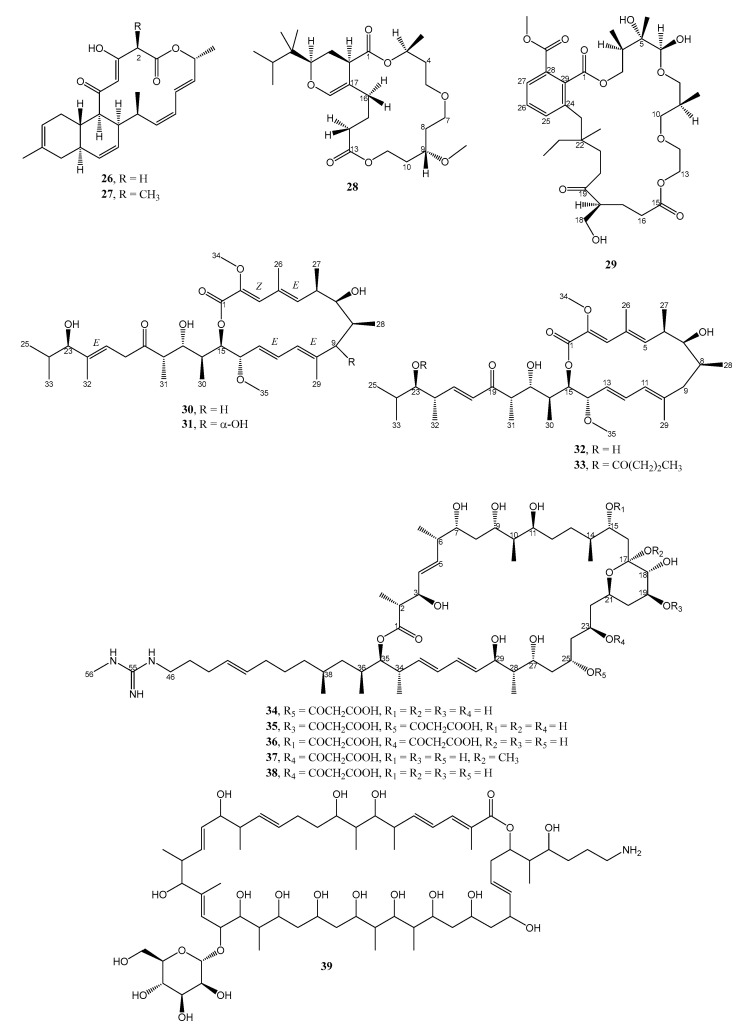
Structures of **26**–**39**.

An undescribed anthracimycin congener, anthracimycin B (**26**) (Figure 3), was obtained together with the previously reported anthracimycin (**27**), from the EtOAc extract of the solid culture (R5A agar) of a marine-derived *Streptomyces cyaneofuscatus* M-169, which was isolated from a gorgonian coral (Order Gorgonacea) collected at 1500 m depth in the Avilés submarine Canyon, Cantabrian Sea [23]. The structure of **26** was determined by ESI-TOF-MS and 1D and 2D NMR spectral analysis as well as by comparison of its NMR data with those of **27 [24]**. Since the optical rotation of **26** has the same sign (levorotatory) and similar magnitude of rotation to those of **27** whose absolute structure was established by X-ray diffraction by Jang et al. [24], the authors concluded that absolute configurations of the stereogenic carbons in **26** were the same as those of **27**. Compounds **26** and **27** were tested against four Gram-positive MSSA and MRSA, vancomycin-sensitive *E. Faecium*, and vancomycin-sensitive *E. Faecalis*, two Gram-negative (*E. Coli* and *K. Pneumoniae*), and *M. tuberculosis*. Compound **26** displayed potent antibacterial activity with MIC values of 0.33–0.65 µM against *S. aureus* MRSA, 10.5–20.9 µM against *S. aureus* MSSA, 0.33–0.65 µM against *E. faecium* VANS and 0.65–1.26 µM against *E. faecalis* VANS while **27** not only exhibited more potent activities against these bacterial strains than **26** but also against *M. tuberculosis* [23]. 

Two unreported macrocyclic polyketides containing dodecahydropyrano-trioxacyclooctadecine dione (**28**) and trioxo-octadecahydro-1*H*-benzo[*o*]tetraoxacyclopentacosine carboxylate (**29**) (Figure 3) were isolated from the EtOAc extract of the agar nutrient culture of a heterotrophic *Gamma-proteobacterium Shewanella algae* MTCC 12715, associated with an intertidal red alga, *Hypnea valentiae*, which was collected from the Gulf of Mannar region in the southeast coast of India. The structures of **28** and **29** were established based on extensive analysis of HRMS, 1D, and 2D NMR spectra. The relative configurations of the stereogenic carbons of the macrocyclic rings were established based on NOESY correlations and supported with molecular mechanistic studies by MM2 force-field calculations by Chem3D Pro (ver-12.0).

Compounds **28** and **29** displayed potential antibacterial activity against methicillin-resistant *S. aureus* MRSA (≥20 mm) and vancomycin-resistant *E. faecalis* VREfs (≥25 mm, 30 μg on disc), whereas the standard antibiotic disc of streptomycin (30 μg) displayed a smaller clearance zone around the disc. In the same manner, **28** and **29** exhibited greater activity (MIC of 3–5 μg/mL) against drug-resistant pathogenic bacteria causing nosocomial infections than chloramphenical (≥6.25 μg/mL) in the microdilution method. The structural attributions and the correlations with bioactivity through various physico-chemical parameters of **28** and **29**, adopted from ChemDraw Ultra ver-12.0 /ACD Chemsketch ver-8.0 databases, revealed that the optimum logarithmic value of the octanol-water coefficient (log Pow), in conjunction with greater electronic parameters and less steric bulkiness of **28** and **29**, could be fundamental for bioactivity. Moreover, in silico docking studies of **28** and **29** exhibited significant inhibition at the allosteric site of the penicillin-binding protein (PBP2a) in *S. aureus,* which supports their in vitro activity profile [25].

Two undescribed bafilomycins, 21,22-en-bafilomycin D (**30**) and 21,22-en-9-hydroxybafilomycin D (**31**) (Figure 3) were obtained from a methanol extract of a GYM solid culture of *Streptomyces* sp. HZP-2216E, isolated from a fresh seaweed, *Ulva pertusa*, which was collected from the Turtle Islet in the South China Sea close to Shanwei City (Guangdong), China. The structure of the compounds was established by extensive analysis of HRMS and 1D and 2D NMR spectra [26]. The geometries of the double bonds were established from the NOE information, while the relative configurations of the stereogenic carbons were determined by NOE information as well as a close similarity of the carbon chemical shift values in the ^13^C NMR spectra of the macrocyclic portion of **30** and bafilomycin D (**32**) [27]. On the other hand, the hydroxyl group on C-23 was deduced as β-oriented since **30** could be prepared from bafilomycin D (**32**) via double bond migration in DMSO solution. In the case of **31**, the α-orientation of the hydroxyl group on C-9 was based on a large coupling constant (*J* = 10.3 Hz) between H-8 and H-9. Compounds **30** and **31** showed growth inhibitory activity against MRSA with MIC values of 12.5 µg/mL for both compounds, while gentamicin, a positive control, showed inhibition with a MIC value of 0.36 µg/mL [26]. Compound **32** was also isolated, together with the undescribed 23-*O*-butyrylbafilomycin D (**33**), from the same bacterium cultured in a 2216E solid medium. Compound **33** exhibited inhibitory activity against MRSA with a MIC value of 7.4 µM, while norfloxacin, a positive control, showed inhibition with a MIC value of 31.3 µM [27].

The complete genome sequence of *Streptomyces* sp. IMB7-145, isolated from a marine sediment sample at a depth of 40 m in Heishijiao Bay, Dalian, China, revealed the presence of the cluster (named *npm*), which spans a 132.4 kbp DNA region and contains 31 individual open reading frames (ORFs), in which nine large genes (*npmA−npmI*) encode a 20-module type I polyketide synthase (PKS). Detailed bioinformatics analysis revealed that the predicted domain and module architecture were in good agreement with the assembly of the polyketide skeleton of niphimycin. The strain was then cultured in the M7 medium (composed of 5 g of glucose, 3 g of peptone, 3 g of beef extract, 2.5 g of starch, 2.5 g of CaCO_3_, 0.001 g of FeSO_4_, 0.001 g of MnCl_2_, 0.001 g of ZnSO_4_, 0.001 g of CuSO_4_, and 0.001 g of CoCl_2_ in 1 L of H_2_O) at 28 °C for 5 days on a rotary shaker at 200 rpm, after which the supernatant and the mycelia were separated. The combined organic extracts of the supernatant and mycelia were fractionated by column chromatography of silica gel and Sephadex LH-20 and purified by a reversed-phase high-performance liquid chromatography (HPLC) to give four niphimycin congeners, niphimycins C−E (**34**–**36**), and 17-*O*-methylniphimycin (**37**) as well as **38** (Figure 3). The planar structures of **34**–**36** were established by HRESIMS, 1D, and 2D NMR spectral analysis. By using *J*-based configurational analysis, the relative configurations of C-2/C-3, C-6/C-7, and C-28/C-29 were established as *anti*, *anti*, and *anti*, respectively. On the other hand, the relative configurations of C-7/C-9/C-10/C-11 were elucidated as *syn/anti/syn* by application of Kishi’s universal NMR database, while the relative configurations of C-17−C-21 of the pyran ring were determined by ^1^H-^1^H coupling constants and ROESY correlations. Additionally, the full absolute configurations were predicted from the ketoreductase (KR) domain analysis of the *npm* gene cluster. It is worth mentioning that the authors have observed that during the isolation process, the previously described **38** [28], also isolated from the extract, could transform to **37** in the MeOH solution in the presence of a trace of acetic acid, suggesting that **37** was an artifact arising from ketalization of **38** with MeOH during the isolation process [29].

Compounds **34**–**37** were tested against representative members of ESKAPE pathogens. Compounds **34** and **38**, with only one malonyl group, showed good antimicrobial activity against methicillin-resistant *S. epidermidis* (MRSE) and *S. aureus* (MRSA), with MIC values of 4–32 µg/mL, but modest activity against vancomycin-resistant *E. faecalis* and *E. faecium* (VRE), with a MIC value of 64 µg/mL. The similar potency of **34** and **38** suggested that the position of the malonyl substituent is not crucial for antibacterial activity. On the contrary, antibacterial activity of **35** and **36,** which contain two malonyl groups, was two-fold less than that of **34** and **38**, suggesting that more malonyl groups in the compounds cause a reduction of antibacterial activity. Moreover, **37** was as active as **34** and **38**, indicating that antibacterial activity was not significantly affected by the ketalization of the hemiketal ring [29].

Interestingly, **34** and **38** showed inhibitory activity against *M. tuberculosis* strain FJ05120 (resistant to isoniazid and rifampicin) and FJ05195 (resistant to isonizaid, rifampicin, streptomycin, and ethambutol) with MIC values of 16 and 32 µg/mL, respectively [28]. 

The extract of the marine actinomycete, *Streptomyces althioticus* MSM3, isolated from samples of the seaweed *Ulva* sp., which was collected in the Cantabrian Sea (Northeast Atlantic Ocean) and cultured in R5A liquid medium at 28 °C at 250 rpm for 6 days, furnished an undescribed desertomycin G (**39**) (Figure 3), a glycosylated macrolide containing a primary amine group. The planar structure of **39** was established by extensive analysis of HRMS, 1D, and 2D NMR spectra, as well as by comparison of its NMR data with those of the previously described desertomycin A [29]. Compound **39** exhibited strong inhibitory activity against various Gram-positive multidrug-resistant clinical pathogens, such as *M. tuberculosis*, *S. Aureus*, *S. Pneumoniae*, *S. Pyogenes*, *Clostridium perfringens*, *C. Urealyticum*, *E. Faecalis*, *E. Faecium*, and moderate antibiotic activity against relevant Gram-negative clinical pathogens, such as *Bacteroides fragilis*, *Haemophilus influenzae,* and *Neisseria meningitidis* [30].

#### 2.1.4. Spirotetronates

**Figure 4:**
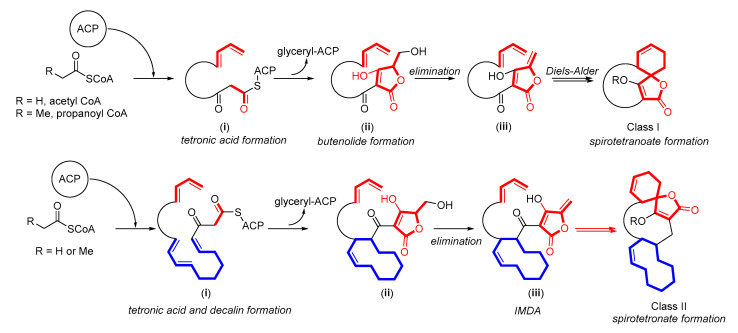
Proposed biosynthetic pathways of spirotetronates in sub-classes I and II.

Spirotetronates are a class of compounds consisting of tetronic acid spiro linked with cyclohexane or cyclohexene moiety and can be divided into two sub-classes: Sub-class I, which consists of spirotetronates connected with a macrocycle, and sub-class II, which contains spirotetronates and macrocycle with an integration of decalin and sometimes oligosaccharide chains [31]. The biosynthesis of spirotetronates of both sub-classes starts with the attachment of acetyl and/or propanoyl CoA to the acyl carrier protein (ACP) to form a chain elongation in type I PKS (PKS I) (Figure 4). In sub-class I, the elongation of the chain is stopped and condensed by a glyceryl-ACP unit after reaching the appropriate length to form tetronic acid (**i**) then undergoes acetylation-elimination to form a butanolide (**ii**). Intramolecular Diels-Alder (IMDA) reaction connects butanolide with a diene portion. The biosynthesis of sub-class II is quite similar to that of sub-class I, but the elongation of the chain in sub-class II incorporates decalin. In addition, glycosylation adds sugar moiety to the structure.

**Figure 5:**
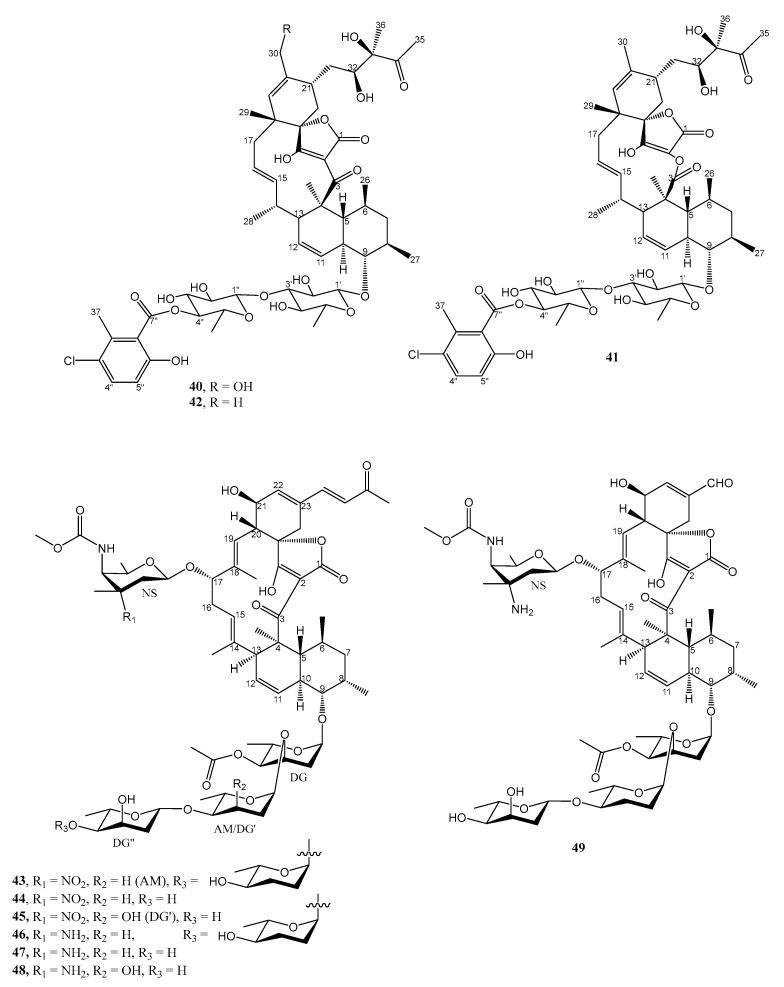
Structures of **40**–**49**.

The acetone extract of a culture of the actinomycete, *Micromonospora* sp. CA-214671, isolated from a marine cave sediment that was collected from the Canary Islands, Spain, and cultured in N4 medium (soluble starch 15 g/L, fish peptone 8 g/L, bacterial peptone 5 g/L, glycerol 6 g/L, KBr 0.2 g/L, CaCO_3_ 2 g/L, and sea salt 10 g/L, and also 3% XAD-16 resin at pH 7.2–7.4) at 28 °C for 7 days, was found to exhibit activity against methicillin-resistant *S. aureus* (MRSA), *M. tuberculosis* H37Ra and *M. bovis*. Bioassay-guided fractionation of this extract led to the isolation of two unreported phocoenamicins B (**40**) and C (**41**), and the previously reported phocoenamicin (**42**) (Figure 5). The planar structures of **40** and **41** were established based on an extensive analysis of 1D and 2D NMR and also HRESIMS spectra, as well as by comparison of their NMR data with those of phocoenamicin (**42**). The relative configurations of their stereogenic carbons were established using NOESY correlations, however, their absolute configurations were not determined. Compounds **40** and **41** exhibited significant activity against MRSA *S. aureus* MB5393, with MIC values of 8–16 and 32–64 µg/mL, respectively, but negligible activity against vancomycin-resistant *E. faecium* (VRE) MB5571 (MIC > 128 µg/mL). Vancomycin, the positive control, showed stronger activity with a MIC value of 2–4 µg/mL [32].

Seven undescribed spirotetronate glycosides, microsporanates A-F (**43**–**48**), and tetrocarcon P (**49**) (Figure 5) were isolated from the acetone extract of the mycelium and of the culture broth absorbed on the XAD-16 resin of *Micromonospora harpali* SCSIO GJ089, isolated from a sediment sample obtained from the northern South China Sea, and cultured in N4 medium (soluble starch 15 g/L, fish peptone 8 g/L, bacterial peptone 5 g/L, glycerol 6 g/L, KBr 0.2 g/L, CaCO3 2 g/L, and sea salt 10 g/L, at pH 7.2–7.4) with 3% XAD-16 resin at 28 °C for 7 days. The structures of the compounds were determined based on extensive analysis of 1D and 2D NMR and also HRESIMS spectra. Compounds **43** and **44** showed moderate inhibitory activity against methicillin-resistant *S. aureus* shhs-A1 (MRSA, a clinical isolate) with a MIC value of 32 µg/mL, while the rest of the spirotetronate glycosides **45**–**49** showed MIC values of >128 µg/mL. Vancomycin, kanamycin, and ampicillin inhibited the same MRSA strain, with MIC values of 2.0, 64, and 1.0 µg/mL, respectively. The results indicated that the type of sugar moieties on C-9 was important for their bioactivities. Nitro sugars showed superior anti-MRSA compared to their amino sugar counterparts. The addition of sugar and C-17, as well as a change of C-23 α,β-unsaturated carbonyl moiety with a formyl group (CHO), have no effect on the anti-MRSA activity [33].

**Figure 6:**
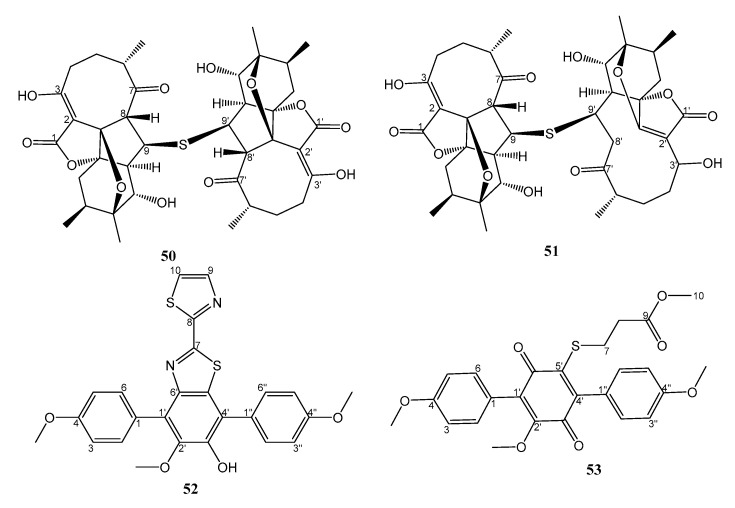
Structures of **50**–**53**.

Abyssomicins are a family of antimicrobial compounds which are generally composed of C19 spirotetronates and often contain a four- or five-membered ring system within their architectures. A majority of abyssomicin analogs exist as monomers, and the only known dimer was abyssomicin J. In order to reanalyze the HPLC dataset of the metabolic profile of *Streptomyces koyangensis* SCSIO 5802, isolated from a sediment collected in the Northern South China Sea, Huang et al. [34] performed a large-scale (60 L) fermentation of the strain SCSIO 5802. Extraction and HPLC separation resulted in the isolation of monomeric abyssomicin congeners with undescribed frameworks (neoabyssomicins) and two undescribed abyssomicin dimers, named neoabyssomicins F (**50**) and G (**51**) (Figure 6). The structures of both compounds were established by HRMS, 1D, and 2D NMR spectral analysis. Key HMBC correlations indicated that the two monomers were connected via a C-9−S−C-9′ thioether bond to form a dimer. In the case of **50**, X-ray crystallographic analysis confirmed the structure and determined the absolute configurations for all stereogenic carbons. Both **50** and **51** showed growth inhibitory activity against three clinical isolates of MRSA (MRSA-shhs A1, MRSA-699, and MRSA-1862) with the same MIC value of 16 μg/mL. Ampicillin (MIC values of 64, 4, 0.125 μg/mL) and vancomycin (MIC values of 0.5, 0.125, and 0.125 μg/mL) were used as positive controls [34].

### 2.2. Amino Acid Derivatives

A number of secondary metabolites produced by bacteria are originated from amino acids. These metabolites can be classified into several structural groups as follows.

#### 2.2.1. Simple Amino Acid Derivatives

*p*-Terphenyls originate from a condensation of two precursors, which are derived from L-Phe/L-Tyr and L-Trp, which is catalyzed by a tridomain nonribosomal peptide synthetase to yield polyporic acid (PPA). Subsequent reduction and dehydration of PPA give terphenyl triol, a *p*-terphenyl scaffold [35].

By using the MS/MS-based Global Natural Products Social (GNPS) molecular networking analysis, two antibacterial *p*-terphenyl derivatives, nocarterphenyls D (**52**) and E (**53**) (Figure 6) were isolated from the EtOAc extract of a liquid culture (1% soluble starch, 1% glucose, 0.5% peptone, 0.2% yeast extract powder, 1% glycerinum, and 0.25% corn flour, pH = 7.0, in seawater) of the marine-derived actinomycete, *Nocardiopsis* sp. HDN154086, isolated from a sediment sample that was collected from the South China Sea. The structure of **52** was elucidated by 1D and 2D NMR and HRMS spectral analysis and was confirmed by single X-ray diffraction analysis using MoKα radiation. The structure of **52** consists of a *p*-terphenyl scaffold with the central benzene ring fused with a 2,2′-bithiazole moiety. On the other hand, the structure of **53** consists of a *p*-terphenylquinone with a methyl proprionate linked to a quinone ring through a sulfur atom [36]. 

Compound **52** displayed antibacterial activity against *Proteus* sp., *B. cereus*, *M. phlei*, *B. subtilis*, *Vibrio parahemolyticus*, and *E. coli*, with MIC values ranging from 1.5 to 6.2 μM, but inactive against MRSA (MIC > 50 µM). On the contrary, **53** showed activity against MRSA, with a MIC value of 6.2 μM, which is 8-fold more active than the positive control, ciprofloxacin (MIC = 50 µM), and also against *Proteus* sp. and *B. subtilis*, with MIC values of 12 μM but inactive against *M. phlei*, *B. cereus*, *V. parahemolyticus*, and *E. coli* [36].

#### 2.2.2. Linear Peptides

Linear peptides can be formed by a direct condensation of two or more amino acid residues to form amide linkages or can be modified to form pyrazinone or diketopiperazine ring. 

**Figure 7:**
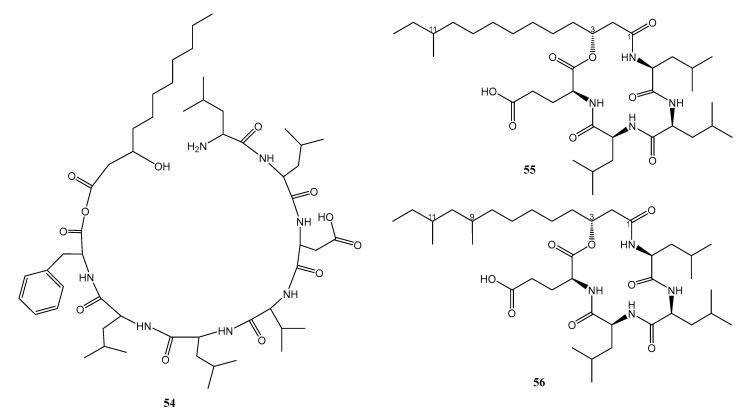
Structures of **54**–**56**.

An undescribed lipopeptide, nesfactin (**54**) (Figure 7), was obtained from the culture extract of an actinomycete, *Nesterenkonia* sp. MSA31, isolated from the marine sponge *Fasciospongia cavernosa*, which was collected from the southwest coast of India [37]. The structure of **54** was elucidated by interpretation of the fragmentation patterns from the liquid chromatography-mass spectrometry (LC-MS/MS). Compound **54** was found to inhibit virulence phenotypes, including the production of hemolysin, protease, lipase, phospholipase, esterase, elastase, rhamnolipid, alginate, and pyocyanin, as well as motility of *P. aeruginosa* strain FSPA02 which is resistant to cefipime, ceftazidime, aztreonam, gentamycin, ampicillin, kanamycin, vancomycin, tetracycline, and streptomycin. By using high-performance thin layer chromatography (HP-TLC) and the reporter assay using CV026.G, it was found that **54** also inhibited the quorum sensing molecule, *N*-acyl-homoserine lactones (AHL), extracted from the culture supernatants of *P. aeruginosa*. Additionally, **54** also inhibited a biofilm formation in *P. aeruginosa*, as observed in a test tube, microtiter plate as well as confocal image analysis. Molecular docking studies showed that the *LasR* protein had binding energy—4.5 kcal/mol. It has two hydrogen-bonding interactions (Ala59 and Lys34 with binding distance of 2.8 and 3.5 Ằ) and one hydrophobic interaction (Ala58:4.01381) against **54** [38].

#### 2.2.3. Cyclic Peptides

Two unreported cyclic lipotetrapeptides, named bacilotetrins A (**55**) and B (**56**) (Figure 7), were obtained from the culture extract of *B. subtilis* 109GGC020, isolated from a sediment sample collected from the Gageocho reef, Republic of Korea. The structures of **55** and **56** were elucidated by extensive analysis of HRESIMS, 1D, and 2D NMR spectra. The absolute configurations of the stereogenic carbons of the amino acids were established by hydrolysis of the lipotetrapeptides, followed by derivatization with Marfey’s reagent and chiral HPLC analysis. The absolute configuration of C-3 of 3-hydroxy fatty acids in both compounds was established as *R* by direct comparison of their specific rotation values with those of the previously reported 3-hydroxy fatty acids [39]. 

Compounds **55** and **56** were tested against several clinically isolated MRSA strains, i.e., ATCC25923, XU212, SA1199B, RN4220, and EMRSA15, by broth dilution method. Compound **55** showed antibacterial activity against ATCC25923, XU212, SA1199B, RN4220, with MIC values of 8, 16, 8, and 32 µg/mL, respectively), whereas **56** was active against ATCC25923, XU212, and SA1199B, with MIC values of 16, 16, and 32 µg/mL, respectively The positive control, norfloxacin, showed MIC values of 16, 16, 64, 2, and 4 µg/mL, against ATCC25923, XU212, SA1199B, RN4220, and EMRSA15, respectively [39].

**Figure 8:**
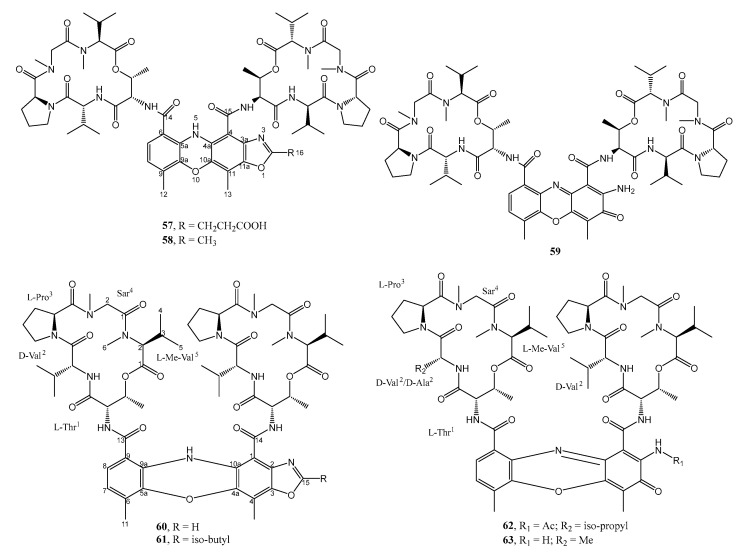
Structures of **57**–**63**.

**Figure 9:**
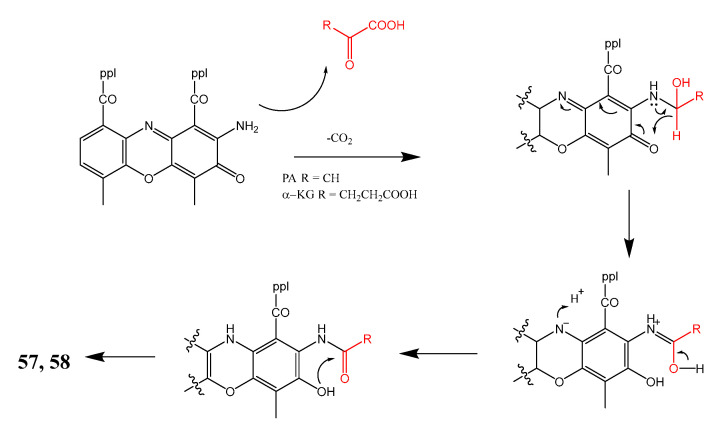
Proposed biogenesis of **57** and **58**.

The liquid culture extract of *Streptomyces* sp. IMB094, isolated from a marine sediment at a depth of 40 m in Heishijiao Bay, Dalian, China, yielded two unreported actinomycin analogs containing a tetracyclic 5*H*-oxazolo[4,5-*b*]phenoxazine chromophore, named neo-actinomycins A (**57**) and B (**58**) (Figure 8). The structures of the compounds were established by extensive analysis of HRESIMS, 1D, and 2D NMR spectra. The absolute configurations of amino acids were determined using advanced Marfey’s method after acid hydrolysis of **57** and **58**. The presence of the phenoxazine ring system was proposed to originate from condensation between the previously reported actinomycin D (**59**) with α-ketoglutarate and pyruvate, respectively, as depicted in Figure 9. The hypothesis of the biosynthesis of **57** and **58** was supported by adding α-ketoglutaric acid and pyruvic acid (1 mg/mL) after the cultivation of *Streptomyces* sp. IMB094, followed by the detection of a 12-fold increase in the production of **57** in α-ketoglutaric acid-supplemented cultures compared to the unsupplemented control, while the yield of both **57** and **58** increased about 6-fold, 24 h after pyruvic acid was added into the cultures.

Compounds **57** and **58** were evaluated for their antibacterial activities against a series of Gram-positive and Gram-negative drug-resistant pathogenic bacteria. Compound **57** displayed moderate antibacterial activities against methicillin-resistant *S. aureus* (MRSA) and vancomycin-resistant *Enterococci* (VRE) strains, with MIC values of 16–64 μg/mL, 64–256-fold less active than actinomycin D (**59**) which was used as a positive control. On the contrary, **58** did not show any antibacterial activity (MIC >128 μg/mL) [40]. 

Four unreported D-type actinomycin analogs, actinomycins D_1_-D_4_ (**60**–**63**) (Figure 8) were obtained from the culture extract of *Streptomyces* sp. LHW52447, which was isolated from a specimen of a marine sponge, *Phyllospongia foliascens*, collected from the Xisha Islands in the South China Sea. The structures of the compounds were elucidated by extensive analysis of HRESIMS, 1D, and 2D NMR spectra. The absolute configurations of the amino acid constituents were established by Marfey’s method. Compounds **60** and **61** represent naturally occurring actinomycins with the oxazole-bearing phenoxazinone chromophore. Compounds **60**–**63** were evaluated for their antibacterial activity against three strains of pathogenic methicillin-resistant *S. aureus* (MRSA), *viz*. P172, P175, and ATCC 33591. Compounds **60** and **61** (with MIC values of 0.125–0.25 µg/mL) displayed higher anti-MRSA activity than **62** and **63** (with MIC values of 0.25–1.0 µg/mL), suggesting that the anti-MRSA activity of this type of compounds might be potentiated by the presence of an oxazole moiety. The positive control, chloramphenicol, showed MIC values of 0.5, 0.5, and 1.0 µg/mL against P172, P175, and ATCC 33591, respectively [41]. 

#### 2.2.4. Alkaloids

**Figure 10:**
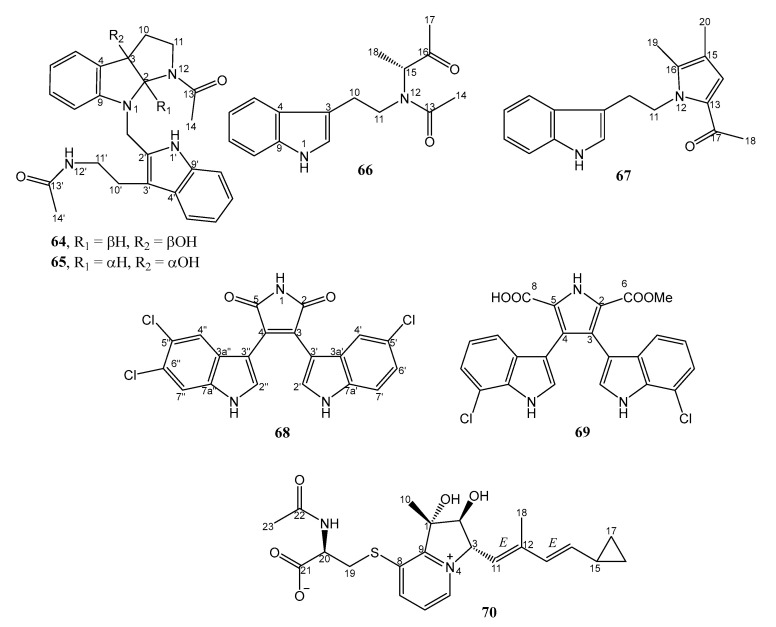
Structures of **64**–**70**.

Four undescribed indole alkaloids, streptoindoles A-D (**64**–**67**) (Figure 10), were isolated from the EtOAc extract of *Streptomyces* sp. ZZ1118, isolated from a gut sample of a marine shrimp (*Penaeus* sp.) in the Zhoushan archipelago, Zhejiang, China, and cultured on the rice medium. Compounds **64** and **65** are enantiomeric bis-indole alkaloids and were separated by HPLC using a chiral column (ChiralCel OJ-RH). The planar structures of **64**–**67** were elucidated by extensive analysis of 1D and 2D NMR as well as HRESIMS data. While the absolute configurations of the stereogenic carbons of **64** and **65** were established by comparison of the calculated and experimental ECD spectra, the absolute configuration of the only chiral carbon (C-15) in **66** was determined by comparison of the experimental and calculated optical rotations. Moreover, the structure of **67** was also confirmed by X-ray analysis [42].

Compound **65** exhibited three times stronger antibacterial activity against MRSA (MIC value of 7 µg/mL) than **64** and **67** (MIC values of 25 µg/mL), while **66** showed no activity at a concentration of 50 µg/mL. The positive control, vancomycin, showed a MIC value of 0.75 µg/mL against MRSA [42].

Two undescribed chlorinated bis-indole alkaloids, dienomycin (**68**) and 6-OMe-7′,7″-dichorochromopyrrolic acid (**69**) (Figure 10), were obtained from the butanone and acetone extracts of a supernatant and a mycelia cake of *Streptomyces* sp. SCSIO 11791, isolated from a sediment sample collected from the South China Sea at a depth of 1765 m and cultured in the ISP-4 medium (containing 0.5% soluble starch, 0.05% yeast extract, 0.1% peptone, 0.1% K_2_HPO_4_, 0.1% MgSO_4_·7H_2_O, 0.2% (NH_4_)2SO_4_, 0.4% NaCl, 3% crude sea salt, 0.2% CaCO_3_, pH 7.4). The structures of **68** and **69** were determined by analysis of HRESIMS, 1D, and 2D NMR spectral data. Compounds **68** and **69** were evaluated for their antibacterial activity against a panel of MRSA, isolated from human patients (MRSA 991, MRSA 1862, MRSA 669 A, and MRSA A2) and pig (MRSA GDQ6P012P and MRSA GDE4P037P). Compound **68** (MIC values = 2 µg/mL) showed stronger anti-MRSA activity than **69** (MIC values = 32–128 µg/mL) against all the MRSA strains but less potent than vancomycin, the positive control, against MRSA 1862, MRSA 669 A, MRSA A2, and MRSA GDQ6P012P (MIC values = 1 µg/mL). Interestingly, **68** showed stronger activity than vancomycin against MRSA GDE4P037P (isolated from pig), with a MIC value of 1 μg/mL compared to 8 μg/mL for vancomycin [43]. 

The undescribed indolizinium alkaloid, streptopertusacin A (**70**) (Figure 10), was obtained from the GYM solid culture extract of *Streptomyces* sp. HZP-2216E, isolated from a fresh seaweed, *Ulva pertusa*, which was collected at the Turtle islet in the South China Sea. The planar structure of **70** was established by extensive analysis of HRESIMS, 1D, and 2D NMR spectral data. The relative configurations of the stereogenic carbons in **70** were established through analysis of proton coupling constants and NOE data. The absolute configuration of the stereogenic carbon of its amino acid moiety (C-20) was determined by Marfey’s method after acid hydrolysis of **70**, while the absolute configurations of C-1, C-2, and C-3 were established by comparison of the experimental and calculated ECD spectra. Compound **70** showed moderate inhibitory activity against MRSA with a MIC value of 40 µg/mL. The positive control, gentamicin, showed a MIC value of 0.36 µg/mL [26].

**Figure 11:**
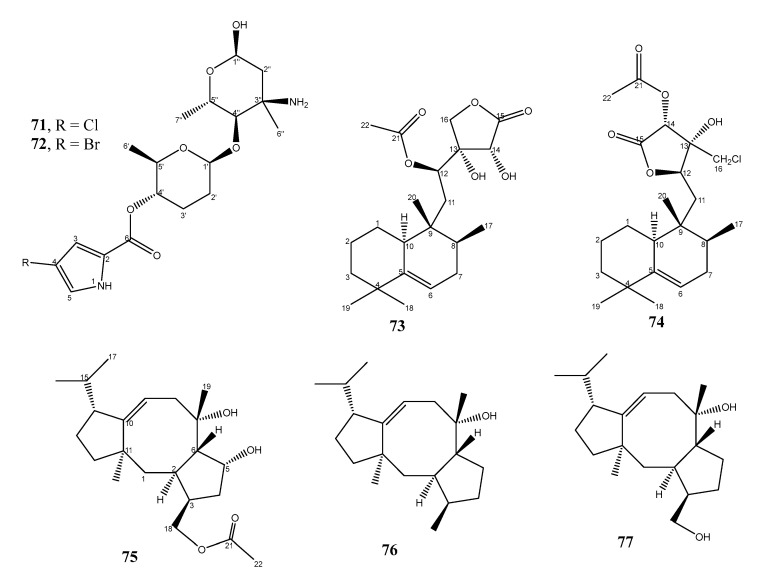
Structures of **71**–**77**.

By using LC-MS-principal component analysis (PCA)-based metabolomics and molecular networking approaches, two antibacterial pyrrole-derived alkaloids, phallusialides A (**71**) and B (**72**) (Figure 11), were obtained from the acetone extract of a bacterium *Micromonospora* sp. WMMA-2495, isolated from a tunicate, *Phallusia nigra*, which was collected in the Florida Keys, and cultured in the artificial medium ASW-A (20 g soluble starch, 10 g glucose, 5 g peptone, 5 g yeast extract, 5 g CaCO_3_ per liter of artificial seawater) supplemented with NaCl and KBr. The structures of the compounds were elucidated by extensive analysis of HRESIMS, 1D, and 2D NMR data. The relative configurations of the stereogenic carbons of **71** were established by a combination of NOE studies, coupling constant analyses, extensive molecular modeling, DFT calculations, and ROESY correlations. Since a comparison of the experimental and calculated ECD spectra allowed the determination of the absolute configuration of only C-4′, the absolute structure of **71** was based on the established relative configurations of the stereogenic carbons relative to C-4′ whose absolute configuration was unambiguously determined. The absolute configuration of the stereogenic carbons in **72** was established by comparison of the Cotton effects observed in its ECD spectrum with those in **71**. Compounds **71** and **72** displayed antibacterial activity against MRSA (ATCC #33591), with a MIC value of 32 µg/mL (vancomycin, the positive control, showed MIC = 0.25 µg/mL). Comparing the structural feature of **71** and **72** with the co-isolated analogs that did not exhibit antibacterial activity revealed that halogenation at C-4 of the pyrrole moiety is crucial to antibacterial activity of **71** and **72**, and the extra sugar moieties in the co-isolated analogs could modulate antibacterial activity [44].

### 2.3. Terpenoid Derivatives

Two undescribed halimane-type diterpenoids, micromonohalimanes A (**73**) and B (**74**) (Figure 11), were isolated from the culture extract of *Micromonospora* sp. WMMC-218, which was obtained from the ascidian, *Symplegma brakenhielmi*, collected in Florida at Stanblum State Park, USA, and cultured in ASW-medium. The structure of **74** was established by HRESIMS, 1D, and 2D NMR spectral analysis. The relative configuration of **74** was established by ROESY, 1D-double-pulsed-field-gradient-spin-echo (DPFGSE)-NOE, and DFT studies. The DFT studies of **74** revealed that stereochemical configurations of **74** were identical to those found in micromonohalimane A (**73**), which was co-isolated and whose absolute structure was determined by X-ray analysis. Compound **74** displayed antibacterial activity against MRSA (ATCC #33591), with a MIC value of 40 µg/mL, while **73** showed a MIC value greater than 200 µg/mL. Vancomycin was used as a positive control and showed a MIC value of 1 µg/mL. Moreover, **74** was found to be a bacteriostatic agent since the MRSA was able to grow in the LB plate in the presence of **74 [45]**.

Three unreported anti-MRSA diterpenoids, *viz*. 18-acetyl-cyclooctatin (**75**), 5,18-dedihydroxy-cyclooctatin (**76**), and 5-dehydroxy-cyclooctatin (**77**) (Figure 11) were isolated from the culture of *Streptomyces* sp. ZZ820, obtained from a soil sample, which was collected from a sea coastal in the East China Sea close to Zhoushan Archipelago, Zhejiang, China, and cultured in the SGYC liquid medium. The planar structures of **75**–**77** were elucidated by interpretation of HRESIMS, 1D, and 2D NMR spectra. The absolute configuration of C-5 in **75** was determined by Mosher’s method, while the absolute structures of **75** and **76** were established by comparison of their calculated and experimental ECD spectra. Since the structure of **77** differs from that of **76** in that Me-18 in **76** was replaced by a hydroxymethyl group in **77**, and both compounds had positive optical rotation values and similar ECD curves, it was assumed that **77** had the same absolute structure as **76**. Compounds **75**–**77** showed moderate anti-MRSA activity with MIC values of 27.45, 24.11, and 29.39 µg/mL, respectively. The positive control, gentamicin, showed a MIC value of 0.91 µg/mL [46].

### 2.4. Miscellaneous

**Figure 12:**
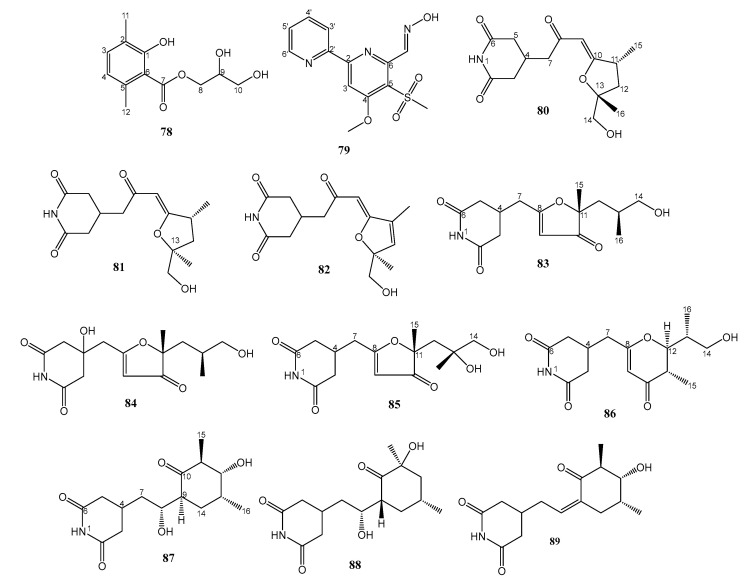
Structures of **78**–**89**.

An undescribed glycerol 1-hydroxy-2,5-dimethyl benzoate (**78**) (Figure 12) was isolated from the culture extract of *Verrucosispora* sp. strain MS100047, obtained from a deep-sea sediment sample collected at the depth 2733 m in the South China Sea, and cultured in the VER01 liquid medium. The structure of **78** was determined by analysis of HRESIMS and 1D and 2D NMR spectra. However, the absolute configuration of C-9 was not determined. Compound **78** showed selective activity against MRSA with a MIC value of 12.5 µg/mL [47]. 

An unreported 2,2′-pyridine containing methyl sulfonyl and carboxaldehyde oxime moieties, named maipomycin A (**79**) (Figure 12), was obtained from a rare actinomycete, *Kibdelosporangium phytohabitans* XY-R10, isolated from the root sediments of a mangrove plant, *Kandelia candel* (L.) Druce, collected from Mai Po Inner Deep Bay Ramsar Site, Hong Kong, China, and cultured in SGTPY medium (17 g sea salts, 5 g starch, 5 g glucose, 1 g tryptone, 1 g peptone, and 1 g yeast extract dissolved in 1 L of distilled water). The structure of **79** was established by analysis of HRESIMS, 1D, and 2D NMR spectra and confirmed by a single-crystal X-ray analysis. Compound **79** did not show antibacterial activity against a panel of Gram-positive and Gram-negative bacteria, except for a weak antibacterial activity against the reference strains and clinical isolate of *Actinobacter baumannii* (MIC = 128 mg/mL). On the contrary, **79** showed an effective inhibitory activity against the Gram-negative bacteria biofilm formation in a dose-dependent manner from 2 to 64 mg/mL. The percentage of biofilm biomass at a minimum biofilm inhibitory concentration (MBIC) was reduced by 84.3% for *A. baumannii*, and 82.6% for *P. aeruginosa* compared to the control. Confocal microscopy analysis showed an increase in the roughness coefficient and surface-to-biovolume ratio of the biofilm, suggesting the heterogeneity and incompleteness of biofilm development caused by **79**. Interestingly, although **79** exhibited weak antibacterial activity, it could efficiently potentiate colistin against *A. Baumannii*. A combination of colistin with **79** resulted in the reduction of the MIC of colistin 4–8 folds. Moreover, **79** also showed a synergistic effect with colistin against *A. Baumannii* but only an additive effect for anti-biofilm activity [48].

The extract of *Streptomyces* sp. ZZ741, isolated from a mud sample in Jintang Island, Zhoushan, China, and cultured on a rice medium, furnished ten undescribed glutarimide antibiotics, streptoglutarimides A−J (**80**–**89**) (Figure 12). The planar structures of **80**–**89** were elucidated based on their HRESIMS data and extensive analysis of their 1D and 2D NMR spectra. The absolute configurations of C-11 and C-13 in **80** were determined as 11*R*, 13*R* by X-ray diffraction analysis and confirmed by comparison of their experimental and calculated ECD spectra. The absolute configurations of C-11 and C-13 in **81** were determined as 11*R*, 13*S* by comparison of their experimental and calculated ECD spectra. Therefore, **80** and **81** are diastereomers. Compound **82** has only one stereogenic carbon (C-13), and its absolute configuration was established as 13*S*, also by comparison of their experimental and calculated ECD spectra. The absolute configurations of the stereogenic carbons in **83**–**85** were determined by comparison their experimental and calculated ECD spectra as well as by ^13^C chemical shift calculations. The absolute configurations of the stereogenic carbons in **86**–**88** were determined by Mosher’s method and by comparison of their experimental and calculated ECD spectra, while the absolute configurations of the stereogenic carbons in **89** were established by NOE correlations and comparison of their experimental and calculated ECD spectra.

Compounds **80**–**89** displayed antimicrobial activities against MRSA, with MIC values ranging from 9–11 µg/mL, while the positive controls, vancomycin, and gentamycin, exhibited MIC values of 0.2 and 0.5 µg/mL, respectively [49].

**Figure 13:**
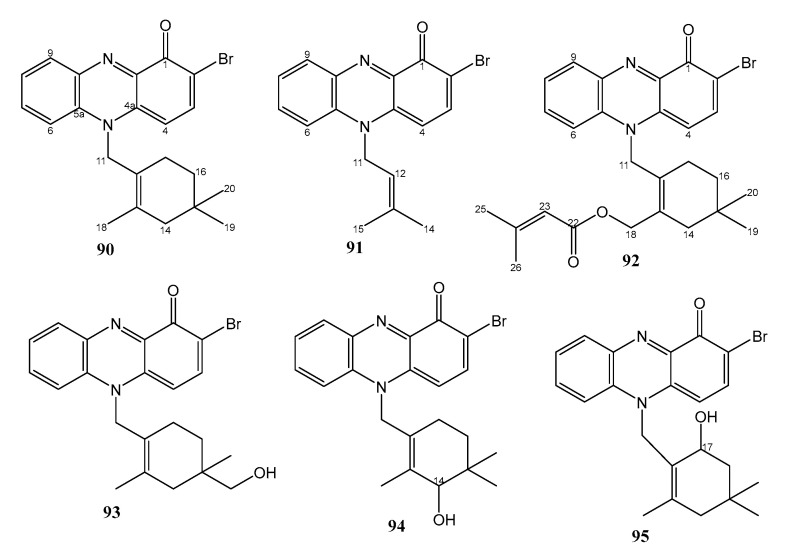
Structures of **90**–**95**.

The unreported *N*-isoprenoid bromo-phenazinone, marinocyanin A (**90**) (Figure 13), was isolated from the culture extracts of the strains CNS-284 (isolated from a sediment sample collected at a depth of 34 m in Palau) and CNY-960 (isolated from a sediment sample collected in the Solomon Islands), while the undescribed marinocyanins B-F (**91**–**95**) (Figure 13) were isolated from the culture extract of the strain CNS-284. Both strains were identified as members of MAR4 clade within the Streptomycetaceae. The planar structures of **90**–**95** were elucidated by HRESIMS, 1D, and 2D NMR spectral analysis. The structure of **90** was confirmed by X-ray crystallographic analysis. The absolute configurations of the stereogenic carbons in **93** and **95** were not determined due to the limited amount of the compounds and their stability [50]. 

Compound **90** strongly inhibited the growth of amphotericin-resistant *Candida albicans*, with a MIC value of 0.95 µM. Compounds **93**–**95** exhibited weaker inhibitory activity against *C. albicans*, with a MIC value of 14.67 µM. Compounds **91** and **92** showed MIC values of 5.79 and 3.90 µM, respectively. The MIC value of the positive control, amphotericin B, was 0.084 µM. Compound **90** exhibited a MIC value of 2.37 µM against *S. aureus*, while **91**–**95** only showed MIC values in the range of 30.71–36.62 µM. The structure–activity relationship indicated that the terpenoid ring system was important for the activity. Modification in the ring system significantly reduced the potencies of the marinocyanins [50].

**Figure 14:**
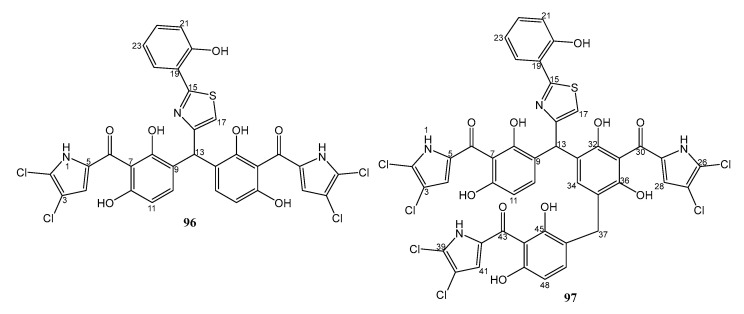
Structures of **96** and **97**.

Bioactivity-guided isolation of the culture extract of *P. aeruginosa* strain 1682U.R.0a.27, isolated from the gill of a giant shipworm, *Kuphus polythalamius*, which was collected in Sultan Kudarat, Mindanao, Philippines, and fermented in R2A medium (0.005% yeast extract, 0.005% protease peptone, 0.005% casamino acids, 0.005% dextrose, 0.005% soluble starch, 0.003% sodium pyruvate, 0.003% K_2_HPO_4_, 0.0005% MgSO_4_, and 2% NaCl) yielded pyoluteorin analogs, mindapyrroles B (**96**) and C (**97**) (Figure 14). The structures of **96** and **97** were established by interpretation of HRMS, 1D, and 2D NMR spectra. Compounds **96** (MIC = 4 µg/mL) displayed more potent antibacterial activity than **97** (MIC = 8 µg/mL) against MRSA strain ATCC 43300 (oxacillin, the positive control, showed a MIC value of 8 µg/mL). Compound **96** also inhibited Gram-negative bacteria, such as *P. aeruginosa* and *E. faecium*, with MIC values of 8 µg/mL. It is interesting to observe that the marinopyrroles, which are coupled by C-C bonds, displayed weaker antibacterial activity than those with the C-N bonds. Within the marinopyrroles, the addition of the hydroxyphenylthiazole moiety led to an increase in antimicrobial activity [51].

## 3. Conclusions

**Figure 15:**
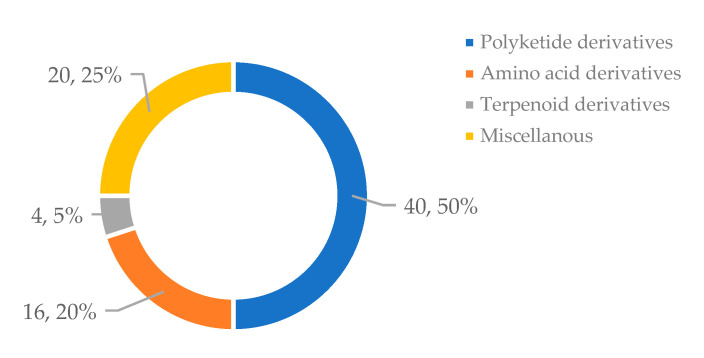
Number of the compounds based on their skeleton-types.

Marine-derived bacteria have been acknowledged as an important source of compounds with unique structures and potential bioactivities. Moreover, the biomass of marine bacteria can be scaled up to an unlimited level in the laboratory for secondary metabolite production. The structures of antimicrobial compounds derived from marine bacteria reported from January 2016 to December 2021 consisted mostly of polyketides, amino acid derivatives, terpenoid derivatives, and also derivatives of mixed origins (Figure 15). As can be seen in Table 1, *Streptomyces* species constitute a major source of bioactive compounds. However, other bacterial taxa, such as *Nonomuraea* sp., *Micromonospora* sp. *Shewanella* sp., *Bacillus* sp., are also found to produce potential compounds with interesting activity against drug-resistant pathogens. Thus, marine-derived bacteria are an excellent source for further exploration to search for novel antimicrobial and antibiofilm compounds against multi-drug resistant pathogens.

## Figures and Tables

**Table 1:** Summary of the compounds with antimicrobial properties against drug-resistant pathogens and their sources.

No.	Bacterial Strain	Source	Compounds and Their Activities	Ref.
1.	*Bacillus subtilis* 109GGC020	Sediment sample from the Gageocho reef, Republic of Korea	**55** and **56** active against MRSA	[39]
2.	*Kibdelosporangium phytohabitans* XY-R10	Root sediments of a mangrove plant, *Kandelia candel* (L.) Druce, from Mai Po Inner Deep Bay Ramsar Site	**79** active against *A. baumanii* as antibiofilm	[48]
3.	MAR4 clade within the Streptomycetaceae strains CNS-284 and CNY-960	Sediment sample from Solomon Islands	**90**–**95** active against amphotericin B-resistant *C. albicans*	[50]
4.	*Micromonospora harpali* SCSIO GJ089	Sediment from Northern South China Sea	**43** and **44** active against MRSA	[33]
5.	*Micromonospora* sp. CA-214671	from the Canary Island	**40** and **41** active against MRSA	[32]
6.	*Micromonospora* sp. RJA4480	Marine sediment from Barkley Sound, British Columbia	**18**–**21** active against MRSA	[20]
7.	*Micromonospora* sp. WMMA-2495	Tunicate, *Phallusia nigra*, from Florida Keys	**71** and **72** active against MRSA	[44]
8.	*Micromonospora* sp. WMMC-218	Ascidian, *Symplegma brakenhielmi*, Florida at Stanblum State Park, USA	**74** active against MRSA	[45]
9.	*Nesterenkonia* sp. MSA31	Marine sponge, *Fasciospongia cavernosa*, from southwest coast of India	**54** against MDR-*P.aeruginosa*	[38]
10.	*Nocardiopsis* sp. HDN154086	Marine sediment from South China Sea	**52** and **53** active against MRSA, *Proteus* sp. and *B. subtilis*	[36]
11.	*Nocardiopsis* sp. strain HB-J378	Deep-sea sediment	**1**–**3** active against MRSA	[12]
12.	*Nonomuraea* sp. strain MM565M-173N2	Deep-sea sediment from Japan trench	**10**–**13** active against MDR-*E. coli* and MDR-*K. pneumoniae*, MRSA, and VRE	[16]
13.	*Pseudomonas aeruginosa* strain 1682U.R.0a.27	Gill tissue homogenate of the giant shipworm, *Kuphus polythalamius*, from Sultan Kudarat, Mindanao, Philippines	**96** and **97** active against MRSA. **96** also active against *P. aeruginosa* and *E. faecium*	[51]
14.	*Shewanella algae* MTCC 12715	Red algae, *Hypnea valentiae*, from the Gulf of Mannar, India	**28** and **29** active against MRSA and VRE	[25]
15.	*Streptomyces althioticus* MSM3	Seaweed, *Ulva* sp., from the Cantabrian Sea	**39** active against *M. tuberculosis*, *S. aureus*, *S. pneumoniae*, *S. pyogenes*, *Clostridium perfringens*, *C. urealyticum*, *E. faecalis*, *E. Faecium*, *B. fragilis*, *H. influenzae* and *N. meningitidis*	[30]
16.	*Streptomyces cyaneofuscatus* M-169	Gorgonian coral from Avilés submarine Canyon, Cantabrian Sea	**26** active against MRSA, MSSA, *E. faecium*, *E. faecalis*	[23]
17.	*Streptomyces koyangensis* SCSIO 5802	Marine sediment from Northern South China Sea	**50** and **51** active against MRSA	[34]
18.	*Streptomyces pratensis* strain NA-ZhouS1	Marine sediment in Zhoushan, East China Sea	**4** and **5** active against *B. stubtilis*, *E. coli*, *K. pneumoniae*, MRSA, *P. aeruginosa*	[13]
19.	*Streptomyces* sp. 1425S.R.1a.1	Mollusk, *Truncatella guerinii*, from Cebu, Philippines	**24** active against MRSA	[21]
20.	*Streptomyces* sp. 182SMLY	Marine sediment	**6** active against MRSA	[14]
21.	*Streptomyces* sp. CA-271078	-	**15** and **17** active against MRSA and *M. tuberculosis*	[18]
22.	*Streptomyces* sp. EG1	Marine sediment	**25** active against MRSA	[22]
23.	*Streptomyces* sp. EG32	Marine sediment from North Coast of the Mediterranean Sea, Egypt	**8** and **9** active against MRSA	[15]
24.	*Streptomyces* sp. HZP-2216E	Seaweed *Ulva pertusa from* Turtle Islet Guangdong, China	**33** active against MRSA	[27]
25.	*Streptomyces* sp. HZP-2216E	Seaweed, *Ulva pertusa*, from Turtle Islet Guangdong, China	**30** and **31** active against MRSA	[26]
26.	*Streptomyces* sp. HZP-2216E	Seaweed, *Ulva pertusa*, from Turtle Islet Guangdong, China	**70** active against MRSA	[26]
27.	*Streptomyces* sp. IMB094	Marine sediment Heishijiao Bay, Dalian, China	**57** active against MRSA and VRE	[40]
28.	*Streptomyces* sp. IMB7-145	Marine sediment at -40 m in Heishijiao Bay, Dalian, China	**34**–**37** active against MRSA, MRSE, and VRE. **34** also active against MDR-TB	[28]
29.	*Streptomyces* sp. LHW52447	Marine sponge, *Phyllospongia foliascens*, in Xisha Islands in the South China Sea	**60**–**63** active against MRSA	[41]
30.	*Streptomyces* sp. MBTI36	Marine sediment	**14** active against MRSA	[17]
31.	*Streptomyces* sp. SCSIO 11791	Deep-sea sediment from South China Sea	**68** and **69** active against MRSA	[43]
32.	*Streptomyces* sp. ZZ1118	Marine shrimp (*Penaeus* sp.) in Zhoushan archipelago, Zhejiang, China	**64**, **65**, and **67** active against MRSA	[42]
33.	*Streptomyces* sp. ZZ741	Sea mud in Jintang Island Zhoushan, China	**80**–**89** active against MRSA	[49]
34.	*Streptomyces* sp. ZZ820	Coastal soil in Zhoushan Archipelago (Zhejiang, China)	**75**–**77** active against MRSA	[46]
35.	*Verrucosispora* sp. strain MS100047	Deep-sea sediment from South China Sea	**78** active against MRSA	[47]

## Data Availability

Not applicable.
